# Elderly hip fracture patients with isolated calf muscle vein thrombosis are more likely to have suffered an intertrochanteric fracture and be hypertensive

**DOI:** 10.1186/s13018-023-04043-6

**Published:** 2023-07-28

**Authors:** Lin Jin, Lei Dong, Yanbin Zhu, Xiuting Li, Zhiyong Hou, Yingze Zhang

**Affiliations:** 1 Department of Orthopaedic Surgery, The 3rd Hospital of Hebei Medical University, Shijiazhuang, 050051 Hebei People’s Republic of China; 2 Orthopaedic Institute of Hebei Province, Shijiazhuang, 050051 Hebei People’s Republic of China; 3 Hebei Institute of Orthopaedic Biomaterials and Technological Innovation, Shijiazhuang, 050051 Hebei People’s Republic of China; 4 Department of Orthopaedic Surgery, Huai’an Hospital of Huai’an City, Huai’an, 223200 Jiangsu People’s Republic of China

**Keywords:** Deep venous thrombosis, Hip fracture, D-dimer, Clinical epidemiology, Diagnostic performance

## Abstract

**Purpose:**

This study aimed to characterize the preoperative deep venous thrombosis (DVT) of lower extremity by locations and evaluate the diagnostic ability of plasma D-dimer level in elderly patients with hip fracture.

**Method:**

This retrospective study reviewed the elderly patients presenting with a hip fracture definitely undergoing surgical treatment and identified those who had preoperative DVT. Data on demographics, lifestyle habits, comorbidities and laboratory indexes were extracted and collected. Four groups were divided by presence and locations of thrombi: non-DVT (controls), isolated calf muscle vein thrombosis (CMVT), deep calf vein thrombosis (DCVT) and proximal DVT group. The comparisons were conducted between either DVT group or the non-DVT group. Receiver operating characteristic (ROC) curve and the area under the curve (AUC) were used for determining the diagnostic ability of D-dimer for each group.

**Results:**

Among 951 eligible patients included, 298 (31.3%) were found have preoperative DVT. Compared to non-DVT group, patients with CMVT had significantly lower albumin and hemoglobin concentration, more likely an intertrochanteric fracture and a higher prevalence of hypertension (*P* = 0.001, 0.006, 0.002 and 0.024, respectively); DCVT group was not observed to be significantly different in terms of any variables (all *P* > 0.05); and proximal DVT group had older age and more patients ≥ 80 years, lower albumin and hemoglobin concentration, higher prevalence of hypertension, more likely an intertrochanteric fracture, extended time from injury to imaging examination and higher age-adjusted Charlson comorbidity index (ACCI) (all *P* < 0.05). D-dimer demonstrated nonsignificant diagnostic ability for CMVT and DCVT, and a significant but poor ability for proximal DVT (AUC, 0.621; 95% CI 0.542–0.710; *P* = 0.011).

**Conclusion:**

Varying characteristics were found among preoperative DVT stratified by locations after elderly hip fractures, and D-dimer level demonstrated no or poor diagnostic ability for DVTs by locations.

*Level of evidence* level III, diagnostic.

*Trial registration statement* Not applicable.

## Introduction

Preoperative deep venous thrombosis (DVT) is prevalent in elderly patients presenting with hip fracture, challenging the health and social care systems. Literature review showed the extremely variable preoperative DVT rate, ranging from 2.6 to 35%, based on population studied, imaging modalities, and the research focus of investigation [1–4]. Due to the potential to propagation proximally to cause a pulmonary embolism (PE), preoperative screening of DVT of lower extremity patients at risk has become a routine practice in most institutions [5].

Studies focusing on the prophylaxis, diagnosis and therapy for DVT in various medical specialties are increasing exponentially during the past years [6–8]. However, few studies focused on examining the characteristics of DVTs according to locations in the traumatic patients, thus not conductive to development of individualized risk profiles and the targeted prophylactic or therapeutic interventions. In addition, it is unknown whether the plasma D-dimer, a most extensively used, inexpensive and readily available index, is applicable to elderly hip fracture patients, a population with multiple susceptible conditions (old age, trauma, comorbidities, declined organ function reserve) for the development of DVT. In a most recent study, Zhang et al. [9] found D-dimer having a very low specificity of 22% in diagnosing preoperative DVT when using the manufacturer’s recommended cutoff value (0.5 mg/L) and suggested use of the age-adjusted cut-off value (age × 0.02 mg/L), albeit with a poor result. Furthermore, these studies did not distinguish between DVTs of different locations when investigating the diagnostic performance of plasma D-dimer level, and possibly the results were equivocal and unpractical.

In this study, we aimed to 1, describe the characteristics of preoperative DVTs stratified by locations and make direct comparisons with the controls; 2, investigate the diagnostic ability of plasma D-dimer for either location of DVTs; and 3, identify the independent risk factors associated with proximal DVT, considering its more clinical importance.

## Materials and methods

### Inclusion and exclusion criteria

The study protocol approval and a waiver of consent informed were obtained from the Ethics Committees of the Third Hospital of Hebei Medical University (No. 2021-041-1), due to the de-identified data used. The study followed the principles outlined in the Declaration of Helsinki.

This study retrospectively reviewed elderly patients who were 65 years or older presenting with an acute hip fracture caused by low-energy trauma and underwent the subsequent surgical treatment in the Third Hospital of Hebei Medical University between January 2020 and December 2021. Patients who had received a duplex ultrasound (DUS) or venography for detection of preoperative DVT of lower extremity were deemed as eligible. The following were excluded: pathological fracture, open fracture, high-energy injury mechanism, subtrochanteric fracture, bilateral hip fracture, time from injury to DUS or venography exceeding 7 days, polytrauma or multiple fractures, neuromuscular disease or lower extremity muscle dysfunction, cancer, hematopoietic diseases, thrombophilia or hematological disorders and previous venous thromboembolism (VTE) or use of anticoagulants within 3 months before injury.

### Definition and classification of DVT

DVT was confirmed by DUS or venography in accordance with the guidelines for diagnosis of DVT proposed by the Chinese Medical Association (3rd edition) [10]. In our institution, patients with hip fracture were considered as high-risk population for DVT and would be administered with routine examination before operation, most shortly after admission. In accordance with the results of screening, prophylactic or therapeutic doses of low-molecular-weight heparin (LMWH) were administered, in combination with the physical or/and mechanical prophylaxis.

According to the presence and locations of DVT, four groups were divided including: (1) non-DVT group where no DVT was detected, as controls; (2) isolated calf muscle vein thrombosis (CMVT) group involving thrombi only occurring at soleal or/and gastrocnemius veins; (3) deep calf vein thrombosis (DCVT) involving anterior tibial veins, posterior tibial veins, peroneal veins, alone or in combination, regardless of whether CMVT was present; (4) proximal DVT group, involving popliteal vein, common femoral vein, superficial femoral vein, deep femoral vein, alone or in combination, regardless of any others.

### Data collection on variable of interest

Data on variables of interest were extracted via inquiring patients' hospitalization medical record system and the laboratory examination system, specifically including demographics (age and gender), fracture type (femoral neck or intertrochanteric fracture), body mass index (BMI), lifestyles (current smoking or alcohol consumption), comorbidities (hypertension, diabetes, heart disease, cerebrovascular disease, renal disease, liver disease and chronic obstructive pulmonary disease (COPD)) and the age-adjusted Charlson comorbidity index (ACCI) [11], albumin level, hemoglobin concentration and plasma D-dimer level. For laboratory indexes, in most cases the results from the fasting blood sample taken after admission, because generally this was the only one before operation; if one patient had multiple laboratory tests for any reasons, the one closest to but before the imaging test for DVT was used.

D-dimer was measured quantitatively using the immunochromatographic assay kit measurement, and was analyzed on Wondfo FS-301 Auto-Immunofluorescence Quantitative Analyzer (Xiamen, China). D-dimer measurement, together with measurement of albumin and hemoglobin level, was carried out within 1 h after blood sample collection in the central laboratory in accordance with the manufactures' instructions.

Current smoking or alcohol consumption was defined as cigarette smoking or alcohol consumption within the last 6 months before the index hip fracture [12]. Comorbidities were available from medical records where patients’ self-reported conditions were documented. In accordance with the criteria suited for Chinese individuals, patients were classified as with normal weight (BMI 18.5–23.9 kg/m^2^), underweight (BMI < 18.5 kg/m^2^), overweight (BMI 24.0–27.9 kg/m^2^) and obesity (BMI ≥ 28.0 kg/m^2^) [13].

### Statistical analysis

Non-DVT group was used as controls, and patients with CMVT, DCVT and proximal DVT as case groups, respectively; the differences between each case group and controls were investigated.

Continuous variables were expressed with mean ± standard deviation (SD) and their normality status was explored by the Shapiro–Wilk test; for variables distributed normally, Student-*t* test was used for between-group comparisons, and for those skewedly distributed, nonparametric Wilcoxon signed-rank test was used. Categorical data were expressed with number and percentage, and *Chi-square* or *Fisher’s exact* test was used for between-group differences, as appropriate.

To examine the potential role of plasma D-dimer in diagnosing preoperative DVT and to determine whether its diagnostic ability varied by locations of DVT, receiver operating characteristic (ROC) curve and the area under the curve (AUC) were performed. In accordance with a classification previously described, AUC of 0.5–0.6 was considered as no diagnostic ability, 0.6–0.7 as poor, 0.7–0.8 as fair, 0.8–0.9 as good and 0.9–1.0 as excellent [14]. If statistical significance was confirmed, the optimal cutoff value was determined with the corresponding sensitivity and specificity.

Given the clinical importance of proximal DVT that it carried higher risk of propagating proximally to cause a PE, we conducted a multivariate logistic regression analysis adjusting for variables that were tested as statistically significant in the univariate analyses. The results were indicated with odd ratio (OR) with 95% confidence interval (CI).

Statistical significance was set as *P* < 0.05. All above analyses were performed using SPSS 26.0 (IBM, Armonk, New York, USA).

## Results

In consistent with our predefined criteria, 951 eligible patients were included, with females in predominance (73.1% and 697/951) and an average age of 79.4 ± 7.6 years (range, 65–102 years). Approximately 90% (89.3%, 849/951) of patients had at least one comorbidity and the average ACCI was 5.2 ± 1.4 (rang, 2–10). There were slightly more intertrochanteric fractures than femoral neck fractures (52.8% vs 47.2%).

Two hundred and ninety-eight patients were found to have preoperative DVT, indicating an incidence of 31.3%. Isolated CMVT took an overwhelming proportion (75.5%), followed by DCVT (13.4%) and proximal DVT (11.1%), corresponding to respective incidence of 23.7%, 4.2% and 3.5%. Compared to the controls (non-DVT group), CMVT group had significantly lower albumin (36.0 ± 4.4 vs 37.2 ± 4.0, *P* = 0.001) and hemoglobin concentration (108.6 ± 17.6 vs 113.7 ± 17.3, *P* = 0.006), more likely an intertrochanteric fracture (60.4% vs 48.5%, *P* = 0.002) and a higher prevalence of hypertension (60.4% vs 51.8%, *P* = 0.024), DCVT group had no significant differences in terms of any variables (all *P* > 0.05), while proximal DVT group exhibited significant differences for multiple variables, i.e., older age (82.8 ± 6.4 vs 79.0 ± 7.7 years in average, and 72.7% vs 55.1% for age of 80 years or older), lower albumin level (33.8 ± 4.2 vs 37.2 ± 4.0 g/L), lower hemoglobin (107.1 ± 15.5 vs 113.7 ± 17.3 g/L), higher ACCI (5.3 ± 1.3 vs 4.9 ± 1.4), more prevalent hypertension (75.8% vs 51/8%), the more intertrochanteric fracture over femoral neck fracture (78.8% vs 48.5%) and extended time to examination for DVT (4.7 ± 5.3 vs 2.1 ± 3.5), with all *P* < 0.05. (Table 1).

**Table 1:** Univariate comparisons between each of DVT groups and non-DVT group

Variable	Non-DVT group (*n* = 653)	Isolated CMVT group (*n* = 225)	P1	DCVT group (*n* = 40)	P2	Proximal DVT (*n* = 33)	P3
Age (year)	79.0 ± 7.7	79.9 ± 7.1	0.317	79.9 ± 7.3	0.867	82.8 ± 6.4	**0.016**
65–79	340 (52.1)	101 (44.9)	0.063	17 (42.5)	0.240	9 (27.3)	**0.005**
≥ 80	313 (47.9)	124 (55.1)		23 (57.5)		24 (72.7)	
Gender			0.207		0.694		0.940
Male	182 (27.9)	53 (23.6)		10 (25.0)		9 (27.3)	
Female	471 (72.1)	172 (76.4)		30 (75.0)		24 (72.7)	
BMI (kg/m^2^)	23.6 ± 3.9	23.6 ± 4.1	1.000	24.9 ± 4.1	0.141	24.0 ± 3.7	0.908
< 18.5	288 (44.1)	17 (7.6)	0.957	0	0.250	0	0.161
18.5–23.9	54 (8.3)	102 (45.3)		17 (42.6)		20 (60.6)	
24.0–27.9	226 (34.6)	75 (33.3)		17 (42.6)		9 (27.3)	
≥ 28.0	85 (13.0)	31 (13.8)		6 (15.0)		4 (12.1)	
Current smoking	69 (10.6)	19 (8.4)	0.361	7 (17.5)	0.173	5 (15.2)	0.407
Alcohol consumption	42 (6.4)	11 (4.9)	0.402	4 (10.0)	0.329	4 (12.1)	0.269
Albumin	37.2 ± 4.0	36.0 ± 4.4	**0.001**	35.9 ± 4.2	0.182	33.8 ± 4.2	** < 0.001**
Hemoglobin	113.7 ± 17.3	108.6 ± 17.6	**0.006**	110.7 ± 18.7	0.635	107.1 ± 15.5	** < 0.001**
ACCI	4.9 ± 1.4	5.4 ± 1.4	0.137	5.2 ± 1.5	0.278	5.3 ± 1.3	**0.043**
D-dimer	5.1 ± 8.9	4.0 ± 4.2	0.202	5.2 ± 4.5	0.999	4.7 ± 5.1	0.987
Heart diseases	153 (23.4)	61 (27.1)	0.267	12 (30.0)	0.344	10 (30.3)	0.365
Hypertension	338 (51.8)	136 (60.4)	**0.024**	23 (57.5)	0.481	25 (75.8)	**0.007**
Cerebrovascular disease	324 (49.6)	115 (51.1)	0.699	26 (65.0)	0.059	17 (51.5)	0.832
Renal disease	39 (6.0)	16 (7.1)	0.543	2 (5.0)	0.227	2 (6.1)	0.983
Diabetes	181 (27.7)	75 (33.3)	0.110	12 (30.0)	0.755	10 (30.3)	0.747
COPD	127 (19.4)	47 (20.9)	0.640	6 (15.0)	0.488	4 (12.1)	0.296
Fracture type			**0.002**		0.271		0.001
Femoral neck fracture	336 (51.5)	89 (39.6)		17 (42.3)		7 (21.2)	
Intertrochanteric fracture	317 (48.5)	136 (60.4)		23 (57.5)		26 (78.8)	
Time from injury to DVT examinatio*n* (days)	2.1 ± 3.5	2.1 ± 3.0	0.981	2.6 ± 3.3	0.336	4.7 ± 5.3	< 0.001

Bold indicated the statistically significant variables

*DVT* deep venous thrombosis, *CMVT* calf muscle vein thrombosis, *DCVT* deep calf vein thrombosis, *BMI* body mass index, *ACCI* age-adjusted Charlson comorbidity index, *COPD* chronic obstructive pulmonary disease

P1, P2 and P3 for comparisons between group 1, group 2, group 3 and controls

**Fig. 1:**
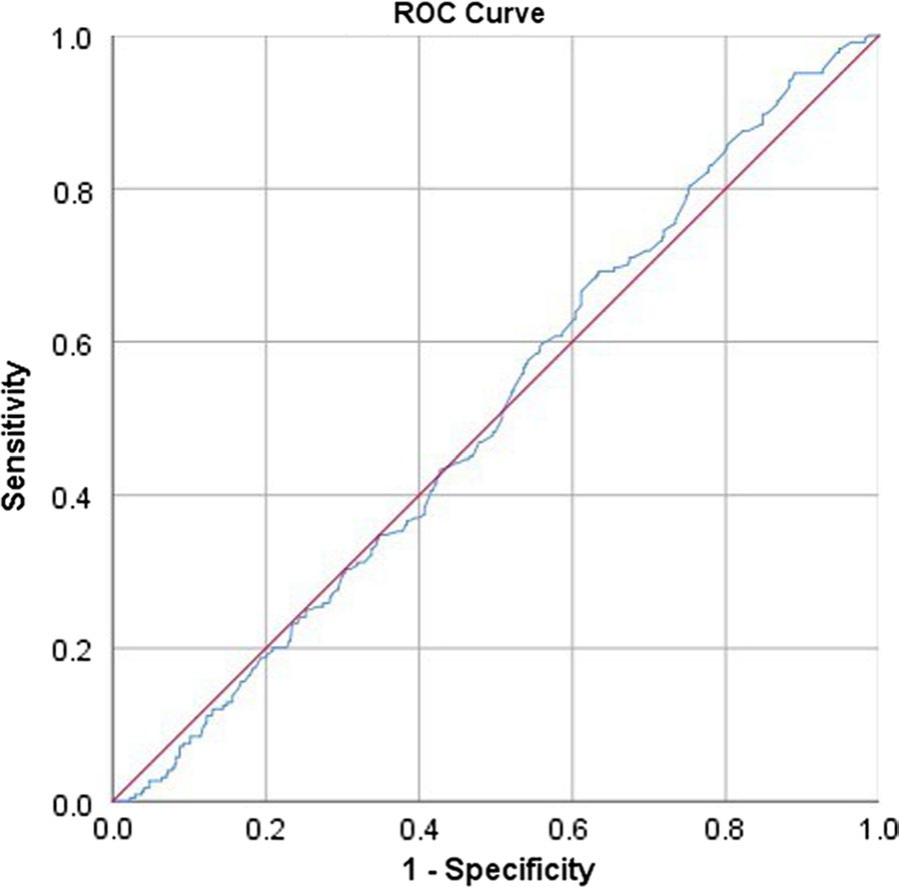
The ROC and AUC analysis for D-dimer in diagnosis of isolated CMVT, demonstrating no ability with AUC of 0.509 (95% CI 0.467–0.552; *P* = 0.675)

**Fig. 2:**
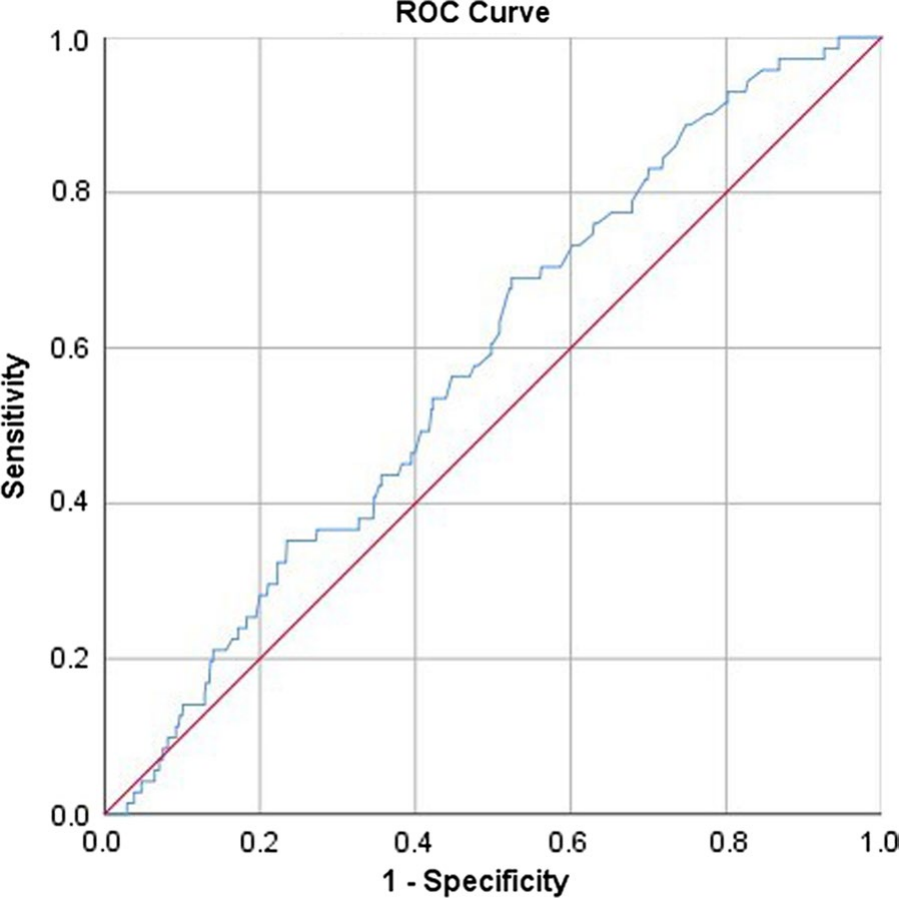
The ROC and AUC analysis for D-dimer in diagnosis of DCVT, demonstrating poor but statistically significant ability with AUC of 0.0.621 (95% CI 0.542–0.710; *P* = 0.011)

**Fig. 3:**
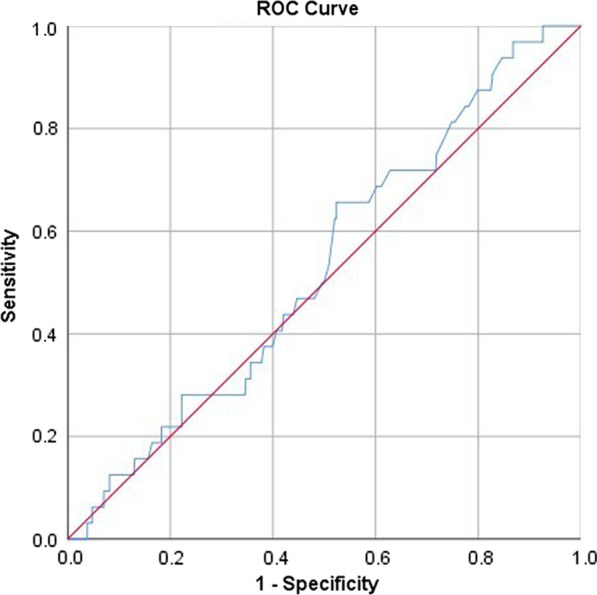
The ROC and AUC analysis for D-dimer in diagnosis of proximal DVT, demonstrating no ability with AUC of 0.532 (95% CI 0.438–0.626; *P* = 0.542)

The ROC analyses and AUC results showed no diagnostic ability of D-dimer either for CMVT or proximal DVT, with AUC of 0.509 (95% CI 0.467–0.552) and 0.532 (95% CI 0.438–0.62) (Figs. 1 and 2); as for DCVT, D-dimer demonstrated a significant but poor diagnostic ability (AUC, 0.621; 95% CI 0.542–0.710) (Fig. 3). The optimal cutoff, i.e., determined when Youden index was maximized, was 2.4 mg/L, with a sensitivity of 0.700 and specificity of 0.467.

Table 2 presented the multivariate analysis results for proximal DVT. The independent risk factors included lower albumin level (OR 1.20; 95% CI 1.07–1.36; *P* = 0.003), hypertension (OR 3.32; 95% CI 1.41–7.79; *P* = 0.006), extended time to imaging for DVT detection (OR 1.14; 95% CI 1.06–1.22) and diagnosis of intertrochanteric fracture over femoral neck fracture (OR 3.75; 95% CI 1.42–9.88).

**Table 2:** The multivariate analysis results for preoperative proximal DVT

Variables	OR and 95%CI	*P*
Albumin (per g/L reduction)	1.20 (1.07–1.36)	0.003
Hypertension (yes vs no)	3.32 (1.41–7.79)	0.006
Time to DVT examination (per day extended)	1.14 (1.06–1.22)	0.001
Intertrochanteric fracture (vs femoral neck fracture)	3.75 (1.42–9.88)	0.003

*OR* odd ratio, *CI* confidence interval, *DVT* deep venous thrombosis

## Discussion

In this study, we found a 31.3% incidence rate of preoperative DVT in elderly patients with hip fractures. Most DVTs were isolated CMVT, taking a proportion of 75.5%. Compared to those without DVT, patients with proximal DVT exhibited more significantly different characteristics, than those with CMVT, while no significant difference was observed between DCVT and non-DVT group. D-dimer demonstrated no or poor diagnostic ability for DVTs stratified by locations. Four risk factors were identified to be associated with increased risk of proximal DVT, including lower albumin level, hypertension, extended time to imaging and diagnosis of intertrochanteric fracture.

In this study, we found a relatively high incidence rate (31.3%) of overall preoperative DVT, which was within the published range (range, 2.6–35%) [1–4], but higher than most ones. The great variation (2.6–35%) was primarily depending on whether isolated CMVT was included. In a most recent study focusing on elderly intertrochanteric fractures where isolated CMVT was excluded, Wang et al. [15] reported an 12.3% rate of preoperative DVT. This figure was slightly higher than 9.8% that was calculated in our study when applying the same conditions, i.e., isolating the intertrochanteric fracture and excluding the isolated CMVT. Inclusion of history of VTE and peripheral vascular disease might explain the additional risk in their study. In another study of preoperative VTE excluding isolated CMVT using indirect multidetector CT venography, Shin et al. [3] reported a 7.7% DVT rate, consistent with our result (incidence for DCVT + proximal DVT, 7.7%, 73/951). To date, the clinical implications of isolated CMVT remain controversial, and increasing evidence suggests that isolated CMVT can be an important source for PE and over 40% of patients with PE had a concurrent isolated CMVT [16], and this subject is ongoing. In two studies where isolated CMVT were included, authors reported the similar rates as ours that were 29.8% and 35.0%, respectively, largely reflecting the acute DVT prevalence in elderly inpatients with hip fracture [1, 2].

As a widely used laboratory index for initial screening of VTE, D-dimer provides an excellent sensitivity (90–100%) and a fair to poor specificity using the manufacturer’s recommended cutoff value (0.5 mg/L) [17]. However, this traditional cutoff value is of extremely limited use for some specific population, like the older (≥ 80 years), because the very low specificity of 0–18% would result in a substantial proportion of false-positive patients [17–19]. In a recent study of a population of elderly hip fracture patients, Zhang et al. [9] attempted to use the age-adjusted cutoff value of D-dimer (age × 0.02 mg/L) to address this issue, but the outcome is unfavorable, i.e., specificity increase to 61% at the expense of significantly lower sensitivity (59%). In the present study, 95.5% (908/951) of patients had a D-dimer ≥ 0.5 mg/L, with an average of 4.8 ± 7.8 mg/L; and even in non-DVT group the corresponding values were 94.5% (617/653) and 3.3 ± 5.3 mg/L. It is clear that the traditional 0.5 mg/L cutoff value had no diagnostic ability, even use of the redefined cutoff by ROC curve provided no or poor diagnostic ability for either DVT subgroup. Therefore, D-dimer is not applicable to elderly patients with hip fractures.

In view of the clinical importance of proximal DVT that was carrying a higher risk of propagation to cause PE or even death, we identified 4 independent risk factors. Intertrochanteric fracture over femoral neck fracture as a risk factor for DVT was first mentioned. It was likely the poorer conditions (older age and medical comorbidities) associated with intertrochanteric fracture that drove the higher risk of DVT, namely intertrochanteric fracture should more be treated as a surrogate for poorer system conditions. This was also the reason that age was no longer statistically significant in the multivariate analyses. Hypertension as a well-established risk factor for VTE in various settings [20, 21], likely reflecting the vascular structural or functional impairment or the unstable hemodynamic status, which was even more prominent after acute traumatic hip fracture. Similarly, the lower albumin level reflected the relatively poor nutritional status or the disrupted physiological balance of protein metabolism responsive of inflammation or trauma (hip fracture), both which can predispose patients to developing thromboembolic problems [22, 23].

Perhaps most strikingly, delay to imaging examination was only statistically significant for proximal DVT, but not for CMVT or DCVT. The exact mechanism underlying this observation is unclear, but may be partly related to the rate of proximal extension of distal DVT [24]. A previous study examining the nature of distal DVT showed that 99% of proximal DVTs had the associated calf vein thrombosis and in over 90% of cases there was a continuous involvement between the proximal and distal veins, suggesting that most thrombi originated in the calf [25]. We believed that delaying to examination allowed the distal thrombus to propagate proximally, thereby increasing the risk of proximal DVT. And in the present study, delay per additional day was associated with 14% increased risk of proximal DVT. Therefore, it is crucial to administer the imaging examination procedure as early as possible and thus the necessary prophylactic or therapeutic interventions.

The strength of this study was the first specifically investigating the diagnostic ability of plasma D-dimer for preoperative DVT stratified by locations in a population of elderly patients with hip fractures. However, some limitations should be noted. First, inherent limitation of retrospective design might have caused inaccuracy in data collection and the effect estimates. Second, we could not capture the accurate time of thrombi formation but only the thrombi status at imaging examination. Future work is needed address this subject using the serial imaging. Third, immobilization (type and duration) of the injured limb would impact on the occurrence of DVT, but the relevant data were not documented in the medical records. Fourth, other confounders or unmeasured covariables would affect the validity of our results and the relationship between risk factors and proximal DVT was associative rather than causative. Therefore, these results should be cautiously treated. Fifth, our results are not applicable to other settings.

In summary, we found a 31.3% rate of preoperative DVT after hip fracture in the elderly patients, with 23.7%, 4.2% and 3.5% for isolated CMVT, DCVT and proximal DVT, respectively. Plasma D-dimer demonstrated no or poor diagnostic ability for DVT by locations, and was not recommended to be used in this population. Four risk factors were identified to be independently associated with proximal DVT and should alert the clinicians to evaluate and stratify the individual risk of proximal DVT.

## Data Availability

All the data will be available upon motivated request to the corresponding author of the present paper.

## Abbreviations

DVT: Deep venous thrombosis
CMVT: Calf muscle vein thrombosis
DCVT: Deep calf vein thrombosis
PE: Pulmonary embolism
BMI: Body mass index
COPD: Chronic obstructive pulmonary disease
ACCI: Age-adjusted Charlson comorbidity index
ROC: Receiver operating characteristic
AUC: Area under the curve
LMWH: Low-molecular-weight heparin
VTE: Venous thromboembolism
OR: Odd ratio
CI: Confidence interval
VTE: Venous thromboembolism
